# Caecal Volvulus Presenting as an Obstructed Inguinal Hernia: A Case Report

**DOI:** 10.7759/cureus.45963

**Published:** 2023-09-25

**Authors:** Viswanathan M S, Vidhya Sree S, Varun M Viswanathan, Sandhya R Palit, Chris Kevin

**Affiliations:** 1 General and Plastic surgery, Employees' State Insurance Corporation (ESIC) Medical College & Hospital, Chennai, IND; 2 General Surgery, Employees' State Insurance Corporation (ESIC) Medical College & Hospital, Chennai, IND; 3 General and Colorectal Surgery, Employees' State Insurance Corporation (ESIC) Medical College & Hospital, Chennai, IND

**Keywords:** right hemicolectomy, hand-sewn anastomosis, intestinal perforation, rare case of intestinal obstruction, caecal volvulus

## Abstract

Caecal volvulus (CV) is an uncommon cause of large intestinal obstruction due to the axial torsion of the caecum, ascending colon, and terminal ileum. We describe the case of a 37-year-old man who presented with bilateral inguinal hernias (the left larger than the right), diffuse abdominal pain, vomiting, difficulty passing stool, and flatus that were comparable to those of an obstructed hernia. Imaging tests revealed a collapsed ascending colon, free fluid collection, and a significantly dilated proximal ileum. An urgent laparotomy showed a perforated, clockwise-twisted caecum that required a right hemicolectomy. Postoperatively, the patient had a good recovery. CV is uncommon, and its symptoms are vague, making diagnosis difficult. For an accurate diagnosis and prompt action, imaging tools and a high index of suspicion are essential. This case serves as a reminder of the significance of taking rare entities into consideration in developing a differential diagnosis of complex abdominal presentations and the necessity for a differential diagnostic approach to choose the most suitable surgical course of action.

## Introduction

Caecal volvulus (CV) is a rare cause of intestinal obstruction, defined by an axial torsion of the caecum, ascending colon, and terminal ileum around the mesenteric vascular pedicles. The incidence of CV is 2.8-7.1 cases per million annually, and it causes approximately 1-1.5% of all intestinal obstructions [1]. Colonic volvulus makes up approximately 10% to 15% of all cases of large-bowel obstructions in the United States and Western Europe. However, its occurrence varies globally, with slightly higher rates observed in regions like India, Africa, and the Middle East, often referred to as the "volvulus belt” [2]. Cultural and dietary factors, influencing intestinal motility, lead to diverse peak ages of presentation across different geographical regions. In India, patients typically present at an average age of 33 years, whereas in Western countries, the average age is 53 years [3].

Depending on the level of preservation of blood supply and the onset of gangrene, mortality ranges from 10 to 40% [3]. Due to its rare occurrence, a definitive diagnosis of CV is often difficult to make [4]. Abdominal computed tomography (CT) scans, barium enema, and colonoscopy are shown to be superior to plain abdominal radiographs in establishing a diagnosis, but they are still not specific enough to make a definitive diagnosis [5]. According to current medical literature, the only certain method to arrive at a diagnosis of CV is through intraoperative confirmation. Since CV has fatal consequences if left undiagnosed, and surgery is the only viable treatment option, a strong suspicion of this clinical condition has to be borne in mind while considering a differential diagnosis. Our article follows the CARE checklist for case reports.

## Case presentation

History

A 37-year-old male patient presented to the emergency department with complaints of diffuse abdominal pain for the past 15 days, which began as an intermittent dull ache and progressed to be constant and increased in severity during the presentation. He had a history of nonprojectile bilious vomiting, refusal of feeds, decreased urine output, and an inability to pass stool and flatus for the past five days. He also had progressive abdominal distension for the past three days.

Clinical examination

On examination, our patient was a moderately nourished, moderately built man. He appeared pale, dehydrated, in acute distress, and febrile. His pulse rate was 120 per minute, and his blood pressure was 94/62 mm Hg. His respiratory rate was 28 per minute. He had a grossly distended abdomen (girth being 102 cm), diffuse tenderness, guarding, and rigidity. Bowel sounds were absent. On examination of the inguinal region, he was found to have bilateral direct inguinal hernia, the left side was larger than the right, with severe tenderness, and he lacked cough impulse, suggestive of possible obstruction and impending strangulation.

Investigations

**Figure 1:**
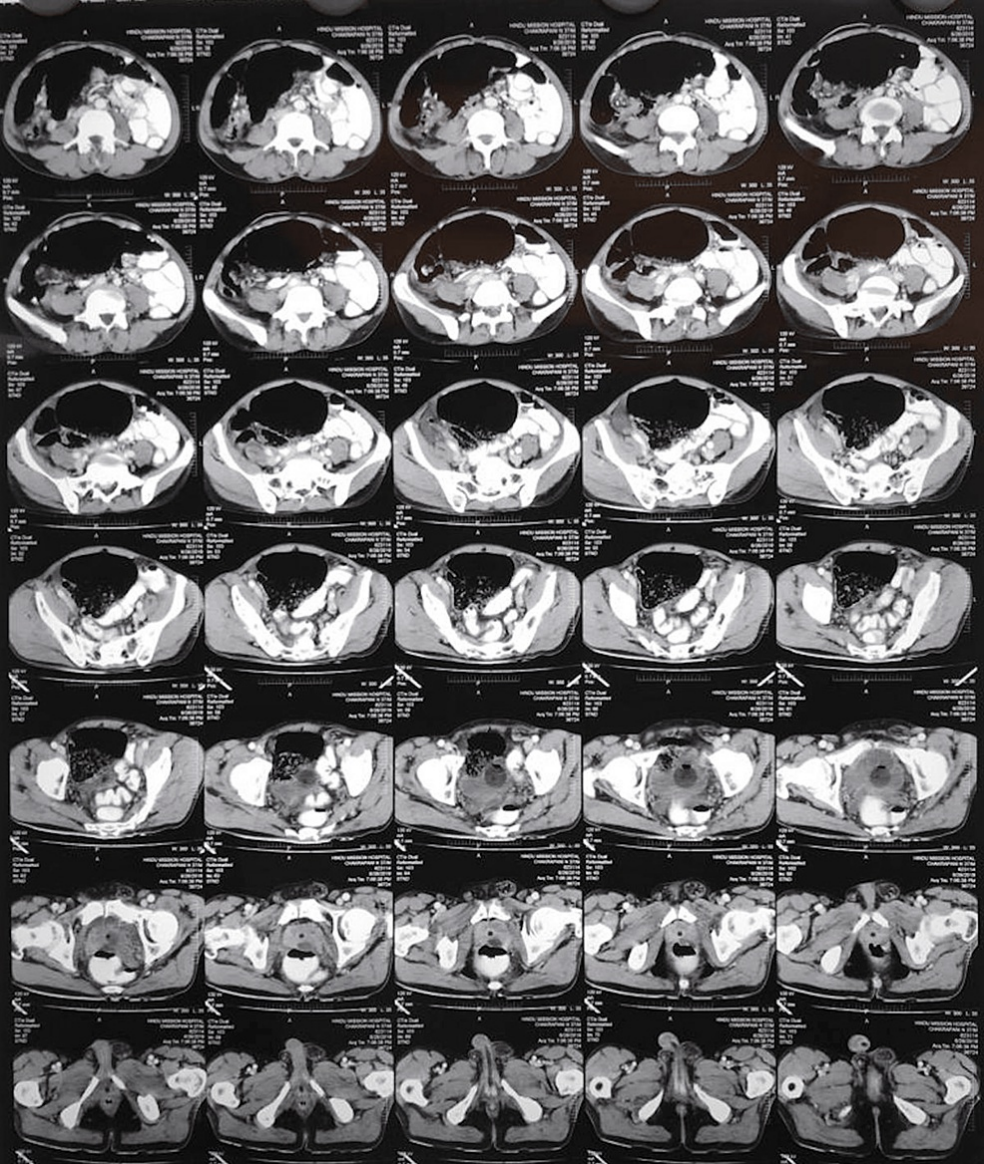
Preoperative abdominal CT image of the patient in favour of caecal volvulus. Gross dilatation of the caecum with a convergence of its two limbs was noted along with proximal dilatation of bowel loops. Mesenteric vessels are seen converging towards the dilated caecum with adjacent mesenteric fat stranding.

Blood investigations revealed leucocytosis (20,683 cells per cubic mm), raised blood urea nitrogen (61 mg/dl), raised lactate levels on arterial blood gas analysis (6.4 mmol/L), dyselectrolytemia (Sr. Na - 119 mmol/L, Sr. K - 5.8mmol/L), and increased liver enzymes (ALT - 103 U/L, AST - 210U/L), pointing towards sepsis with a qSOFA score of 2. Abdominal X-ray showed dilated small bowel loops with air-fluid levels. The patient had presented to us with the following imaging investigations done elsewhere. Abdomino-pelvic ultrasonography reports revealed grade I prostatomegaly, a simple prostatic cyst, and free fluid. A CT scan report of the abdomen and pelvis revealed that the patient had a bilateral direct inguinal hernia, a grossly dilated caecum with the convergence of its two limbs, and proximal dilatation of bowel loops with mesenteric vessels seen converging towards the dilated caecum along with adjacent mesenteric fat stranding. There was a mild dense free fluid collection noted in the peritoneal cavity suggesting particulate ascites (Figure 1). 

Due to the poor general condition of the patient, repeat imaging was not attempted and the reports were discussed with the in-house radiologist, who also confirmed the same findings.

Preoperative findings and management

With such findings, a clinical diagnosis of obstructed inguinal hernia with features of strangulation with possible perforation and peritonitis was suspected. The patient was admitted to the Surgical Intensive Care Unit, resuscitated with IV fluids, and started on broad-spectrum intravenous antibiotics.

**Figure 2:**
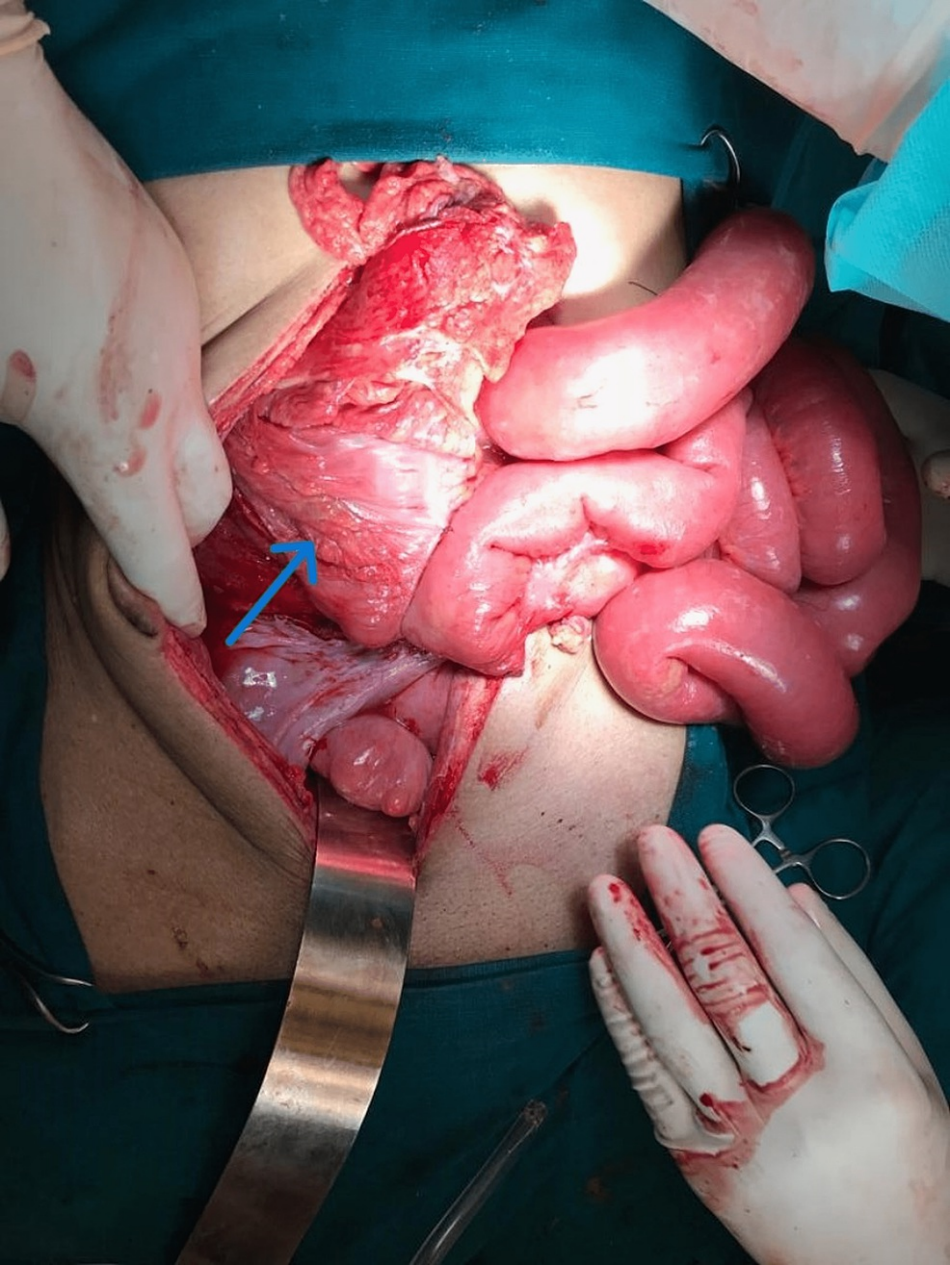
The phlegmon present in the right iliac fossa. The blue arrow indicates the mass in the right iliac fossa with the overlying plastered omentum.

**Figure 3:**
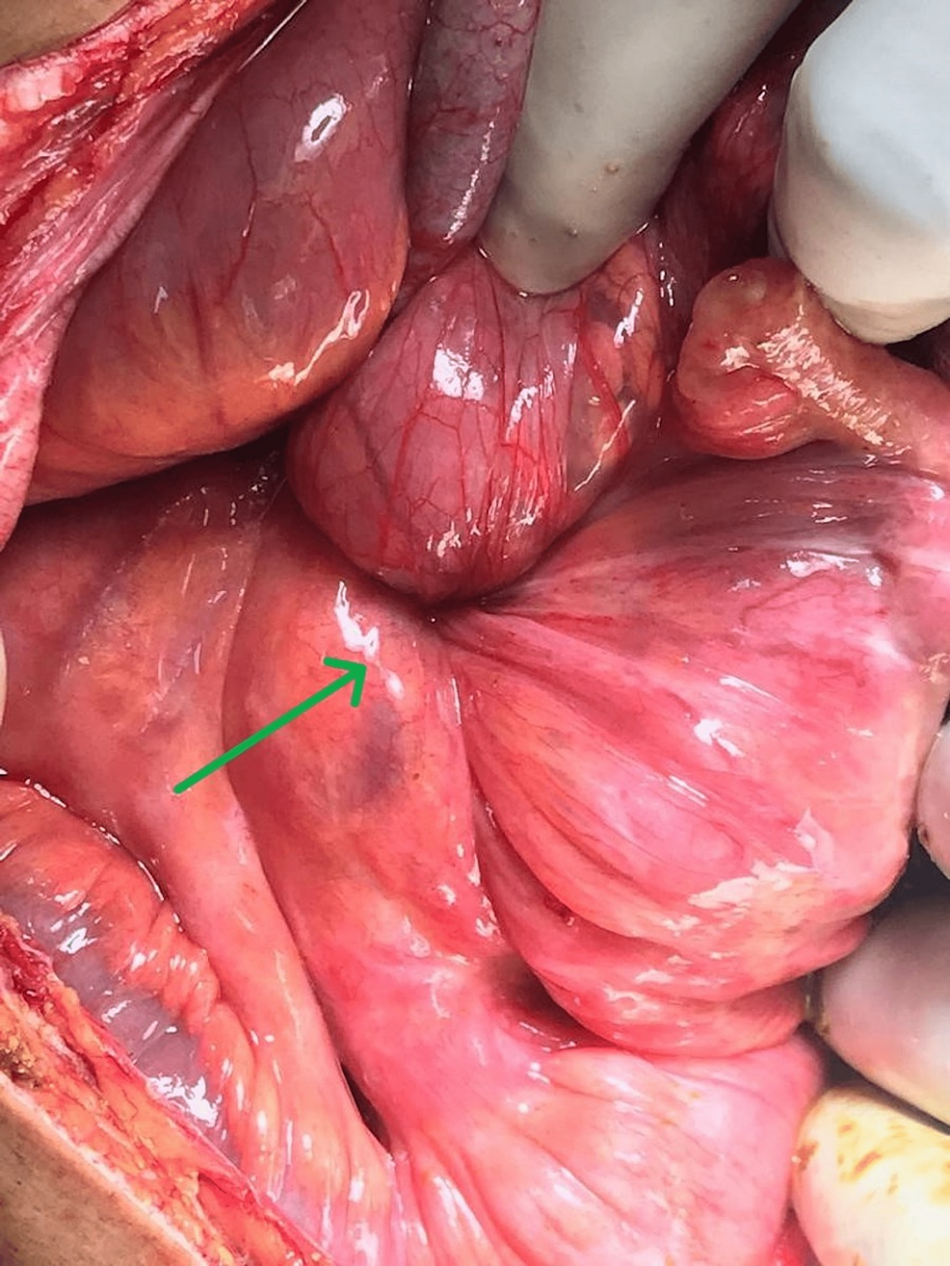
Caecum along with its mesocolon twisted clockwise around its base. The green arrow indicates the twisted caecum with the adjacent ascending colon with the mesentery along its longitudinal axis.

After obtaining informed consent, in view of peritonitis, the patient was taken up for emergency laparotomy. Under anaesthesia, it was noted that the left inguinal hernia had reduced spontaneously. A vertical midline laparotomy incision was made to find 1.5 litres of purulent and foul-smelling peritoneal fluid containing bile and partially formed faeces. There was a phlegmon (Figure 2) present in the right iliac fossa; on blunt dissection of the plastered omentum and the congested terminal ileal loops and releasing the adhesions, the mass was found to be the caecum along with its mesocolon twisted clockwise around its base (Figure 3).

Untwisting the caecum revealed a 3 x 2cm perforation from which the contents were leaking resulting in peritonitis. Right hemicolectomy was done followed by end-to-end ileo-colic stapler anastomosis. Thorough peritoneal lavage was given with 6 litres of normal saline. Abdominal drains were kept in both the flanks and the wound was closed in layers. The patient developed basal atelectasis on the first postoperative day, managed with nebulization and chest physiotherapy. The ileus lasted for 48 hours, and the bowel functions returned on the third postoperative day; hence, the patient was allowed orals. His blood parameters returned to normal limits by the fourth postoperative day. The drains were removed on the eighth postoperative day after observing the reduction in the output (<50ml for three consecutive days). He was then discharged and reviewed on the 14th postoperative day and skin staples were removed. On his third-month follow-up, the patient did not have any other complaints and had a healthy healing scar.

## Discussion

A volvulus is defined as an axial twist of the mesentery of the alimentary canal [4,5]. It can happen anywhere across the digestive tract, including the stomach, gall bladder, and small intestine, but frequently manifests in the colon [6]. Blood flow to the affected intestine may be impeded, leading to ischemia, if the mesentery is further twisted tightly or intestinal dilation is excessive. Volvulus carries a high risk of morbidity and mortality and can quickly develop into bowel ischaemia, necrosis, and perforation if not identified and treated early [6,7]. There are three distinct types of CV: I, II, and III. The most frequent types, I and II, account for 80% of instances and are produced by torsion, whereas type III, which is caused by caecal bascules (the caecum folding upward), accounts for the remaining 20% of cases [8].

CV commonly strikes women under the age of 60 more frequently. This has been attributed to hysterectomies and pregnancy because pelvic surgery may make the caecum more mobile or induce adhesions about which the caecum can rotate [7]. Pregnancy has been a known association because the gravid uterus can lengthen the caecal mesentery [6]. Clinical studies have found that a significant proportion, ranging from 23% to 53%, of patients diagnosed with CV have a history of prior abdominal surgery. The theory suggests that postoperative adhesions play a role in creating fixation points and a pivot for the mobile right colon, thus facilitating the development of CV [3].

Constipation, nausea, vomiting, abdominal cramping, and intermittent abdominal distention are all symptoms of CV. However, in people with "mobile caecum syndrome," these symptoms may resolve on their own. Acute vascular compromise from a CV can cause colonic necrosis and perforation [8-11]. In cases of CV, approximately 25% to 35% of patients experience an emergency presentation characterized by clinical indicators such as peritonitis or shock due to ischemia or perforation [2]. The prognosis is worse when the patient has peritonitis and shock as colonic necrosis and perforation have likely already happened as a result of delayed treatment [3]. Although crucial, laboratory testing only serves to highlight intestinal obstruction, bowel necrosis, and/or sepsis rather than the actual diagnosis. They should aid in directing patient care by raising diagnostic scepticism and highlighting the overall gravity of the patient's disease process. 

In a review of 103 CV cases, Swenson and colleagues found that abdominal X-rays provided suggestive or conclusive evidence of caecal and sigmoid volvulus in 42% and 81% of patients, respectively [9].

Barium enema, a traditional diagnostic tool, boasts an 88% accuracy rate for acute cases. It also allows for occasional volvulus reduction and can identify coexisting issues contributing to volvulus. Previously, barium enema was the go-to imaging method, but abdominal CT is now preferred for its growing use and accuracy in diagnosing acute CV, showcasing characteristic signs like the 'coffee bean,' 'bird beak,' and 'whirl.' This shift is especially pertinent for critically ill patients with advanced obstruction, suspected perforation, or gangrenous bowel, as barium enema may not be suitable. Due to its limited success rate, the risk of colonic perforation, and the possibility of delaying surgical intervention in cases of unsuccessful reduction, using colonoscopy as an initial diagnostic approach or treatment for CV is generally not advised unless performed by a highly skilled physician [3].

In a retrospective analysis of 50 instances of sigmoid volvulus and 53 instances of CV conducted by Swenson and colleagues, CT scans performed without rectal contrast demonstrated an average diagnostic accuracy of 77% [9].

CV patients are virtually always managed surgically. Current surgical choices for CV encompass manual detorsion, caecopexy, caecostomy, and open or laparoscopic colectomy [3]. Since this condition is rare, there are no prospective treatment trials to offer specific guidance. However, there is a consensus that when gangrenous changes and perforations are present, the nonviable intestinal segments should be removed, and the healthy bowel should be anastomosed [3]. Due to recent advancements in laparoscopic technology, laparoscopic colon resections are gaining popularity. Considering the physiological and postoperative benefits of laparoscopy compared to open surgery and the ongoing growth in laparoscopic gastrointestinal procedures, it is likely that laparoscopic right colectomy and caecopexy may become the primary treatment methods in the near future [3,11].

CV can be reduced nonoperatively (e.g. by colonoscopy or contrast enema); however, this method is rarely effective (5%) and may result in perforation [10-12]. Additionally, 20% to 25% of patients who have nonoperative reduction may miss developing colonic necrosis, and such patients may develop colonic perforation [8]. Reported operative mortality varies between 0% and 30%, and recurrence rates range from 0% to 40% in cases where nonresectional treatment is administered [3].

We believe that the most suitable surgical approach for an individual patient should be decided by the surgeon, considering factors such as their surgical skill, the patient's overall health, the condition of the affected intestines, potential complications during surgery, and the likelihood of volvulus recurring.

## Conclusions

CV, being an uncommon reason for large intestinal obstruction, which can even result in the deadliest of consequences, was masked by a less notorious presentation of inguinal hernia in our patient. The index presentation and the subsequent progress of the patient, keeping in mind his imaging findings, had our diagnosis inclined towards a Richter's hernia. This case report is to emphasize the importance of the timely decision to open the abdomen and also though not done in our patient, not to shy away from the placement of stomas on a temporary basis if and when necessary depending on the condition of the bowel as well as the general condition of the patient. 
